# Identification of an Immune Signature Predicting Prognosis Risk and Lymphocyte Infiltration in Colon Cancer

**DOI:** 10.3389/fimmu.2020.01678

**Published:** 2020-09-03

**Authors:** Xinyu Li, Dacheng Wen, Xiaokang Li, Chunli Yao, Wei Chong, Hao Chen

**Affiliations:** ^1^Department of Gastrointestinal Oncology, Shandong Cancer Hospital and Institute, Shandong First Medical University and Shandong Academy of Medical Sciences, Jinan, China; ^2^Department of General, Visceral, and Transplant Surgery, University Hospital, Ludwig Maximilian University of Munich, Munich, Germany; ^3^Department of Gastrointestinal Nutrition and Hernia Surgery, The 2nd Hospital of Jilin University, Changchun, China; ^4^Department of Dermatology, Jinan Central Hospital, Cheelo College of Medicine, Shandong University, Jinan, China; ^5^Department of Dermatology, The 2nd Hospital of Jilin University, Changchun, China; ^6^Department of Gastrointestinal Surgery, Shandong Provincial Hospital Affiliated to Shandong First Medical University, Jinan, China; ^7^Department of Gastrointestinal Surgery, Shandong Provincial Hospital, Cheeloo College of Medicine, Shandong University, Jinan, China; ^8^Key Laboratory of Engineering of Shandong Province, Shandong Provincial Hospital, Jinan, China; ^9^Clinical Research Center of Shandong University, Clinical Epidemiology Unit, Qilu Hospital of Shandong University, Jinan, China

**Keywords:** colon cancer, immune signature, tumor mutation load, immune infiltration, significantly mutated genes

## Abstract

Increasing studies have highlighted the effects of the tumor immune micro-environment (TIM) on colon cancer (CC) tumorigenesis, prognosis, and metastasis. However, there is no reliable molecular marker that can effectively estimate the immune infiltration and predict the CC relapse risk. Here, we leveraged the gene expression profile and clinical characteristics from 1430 samples, including four gene expression omnibus database (GEO) databases and the cancer genome atlas (TCGA) database, to construct an immune risk signature that could be used as a predictor of survival outcome and immune activity. A risk model consisting of 10 immune-related genes were screened out in the Lasso-Cox model and were then aggregated to generate the immune risk signature based on the regression coefficients. The signature demonstrated robust prognostic ability in discovery and validation datasets, and this association remained significant in the multivariate analysis after controlling for age, gender, clinical stage, or microsatellite instability status. Leukocyte subpopulation analysis indicated that the low-risk signature was enriched with cytotoxic cells (activated CD4/CD8^+^ T cell and NK cell) and depleted of myeloid-derived suppressor cells (MDSC) and regulatory T cells. Further analysis indicated patients with a low-risk signature harbored higher tumor mutation loads and lower mutational frequencies in significantly mutated genes of *APC* and *FBXW7*. Together, our constructed signature could predict prognosis and represent the TIM of CC, which promotes individualized treatment and provides a promising novel molecular marker for immunotherapy.

## Introduction

Colon cancer (CC) is one of the most common cancers and remains one of the leading causes of cancer death worldwide (1). Despite continuous achievements in early CC detection, treatment, and management leading to reductions in the incidence and mortality, 30–50% of patients develop recurrence or metastasis within five years of treatment (2). Therefore, in addition to the current clinical and pathological factors for determining the disease prognosis and patient survival, reliable and robust new molecular markers are urgently needed to improve the personalized therapy for CC patients.

Numerous studies have recently highlighted the effects of the immune microenvironment on cancer tumorigenesis, development, and metastasis (3–5). Indeed, assessment of the enrichment of tumor-infiltrating lymphocytes (TIL) was demonstrated to be an important complement role to the TNM staging system for relapse and mortality prediction in CC (6–8). Besides, recent immunotherapies targeting specific immune checkpoints such as PD-1/L1 have demonstrated a remarkably durable response in CC treatment (9, 10). Patients with certain histopathologic patterns, such as intratumoral infiltration by cytotoxic lymphocytes and tumor neoepitope burden, have also been reported with a better clinical prognosis (11–13). Conventional methods for detecting the tumor immune infiltrate, such as flow cytometry or immunohistochemistry (IHC), cannot comprehensively evaluate the immune effects due to the limitation of the number of immune markers. As an alternative, continuously accumulating transcriptomics data provides an ideal resource for large-scale immune landscape analysis (14). However, there has been no appropriate signature that can systematically evaluate the tumor immune micro-environment (TIM) based on immune-related genes and predict the patients’ survival or response to immunotherapies of CC patients. Therefore, it’s essential to develop a reliable immune signature on the basis of a comprehensive list of immune-related genes to represent the immune status of TIM and have the prognostic ability of CC.

In this study, we concentrated on constructing an immune signature with survival prediction and lymphocyte infiltration estimation ability based on the comprehensive list of immune-related genes curated from The Immunology Database and Analysis Portal (ImmPort) database (15). The microarray data from the gene expression omnibus database (GEO) database and RNA sequencing (RNA-seq) data from the cancer genome atlas (TCGA) database were used for analysis and validation. We then evaluated whether this signature was associated with survival outcomes and clinicopathological factors in the CC subgroups. Furthermore, we tried to figure out the relationship between the signature and tumor immune-related indexes including TIL and tumor mutation load (TML) in CC. And finally, we evaluated the effects of this risk signature in identifying the immune responders from immune check-point inhibitors (ICI) therapy. Findings gleaned from this study may be valuable for predicting patients’ prognosis and guiding immunotherapy treatment for CC.

## Materials and Methods

### Publicly Attainable Expression Datasets and Immune-Related Genes

Gene expression data and clinical features of CC samples were retrospectively collected from publicly available datasets at the NCBI GEO database^1^ and at TCGA^2^. The selection criteria of CC datasets were adopted from the workflow of the Dai et.al study (16). A total of 1430 patients were enrolled for analysis, including GSE39582 (*N* = 557) (17), GSE17538 (*N* = 200) (18), GSE37892 (*N* = 130) (19), and GSE33113 (*N* = 90) (20), and TCGA (*N* = 453). The GSE14333 (21) dataset was excluded from this analysis owing to the fact that its probe cell intensity (CEL) files overlapped extensively with the GSE17538 series. Among these cohorts, GSE39582 was the largest set consisting of 557 CC samples, and hence, it was marked as a discovery series and used for constructing the gene signature. Considering the small sample sizes of the GSE17538, GSE33113, and GSE37892 cohorts, and the fact that they shared the same microarray sequencing platform (Affymetrix HG-U133 plus 2.0), we integrated the three datasets into a combined large cohort and regarded this as external validation. The ComBat method from the SVA R package was used to remove the batch effects among different GEO datasets (16). The clinical and survival information of the included datasets was summarized in Supplementary Table S1. An immunotherapeutic cohort of advanced urothelial cancer (IMvigor210 cohort) treated with atezolizumab (anti-PD-L1 McAb) were utilized to further validate the efficiency of the immune risk signature (22). The detailed clinical annotations and complete gene expression profile of the anti-PD-L1 cohort were obtained from http://research-pub.gene.com/IMvigor210CoreBiologies. The comprehensive list of immune-related genes, containing a total of 1508 genes, was downloaded from the ImmPort database^3^ (15).

### Tumor Mutational Load and Neoantigen Analysis

The somatic mutational profile of colon adenocarcinoma (COAD) in the Cancer Genome Atlas (TCGA) mutation annotation format (MAF) were downloaded from Genomic Data Commons (GDC)^4^. Non-synonymous mutations counts were recognized as TML and used for investigating the relationship with the immune signature. Non-synonymous mutations included splice site mutation, missense mutation, nonsense mutation, in-frame mutation, and frameshift mutation. Neoantigens of COAD were collected from previously published studies (23). The antigen peptides resulting from non-synonymous mutated HLA sequences with predicted binding affinities below 500 nM are defined as neoantigens (24).

### Gene Set Enrichment Analysis

The R packages limma (25) were used to evaluate differential expression of more than 21,000 genes in samples with different risk groups. To specify, the expression data were background corrected and quantile normalized and probe sets were summarized using RMA with the affy R package. Subsequently, the normalized expression data were then fed into lmFit and eBayes functions to calculate the differential statistics with the limma package. The logFC produced by limma was used as an input to perform gene set enrichment analysis (GSEA) (26) against the REACTOME reference gene set (MSigDB database v7.1). The fast GSEA algorithm implemented in the Bioconductor R package fgsea was used.

### Immune Cell Infiltration Estimation With ssGSEA

The relative infiltration of 28 immune cell types in the CC tumor microenvironment were quantified by the single sample gene set enrichment analysis (ssGSEA) (27). Special feature gene panels for each immune cell subset were curated from a recent research (4, 28). The relative abundance of each immune cell type was represented by an enrichment score in the ssGSEA analysis. The ssGSEA score was normalized to unify distribution from 0 to 1 for each immune cell type. The bio-similarity of the immune cell filtration was estimated by multidimensional scaling (MDS) and a Gaussian fitting model.

### Quantify the Immunotherapy Response Predictor: Immunophenoscore

The superior immune response molecular marker, Immunophenoscore (28), was used to characterize the intratumoral immune landscapes and the cancer antigenomes. The scoring scheme was created from a panel of immune-related genes belonging to the four clusters: major histocompatibility complex (MHC)-related molecules, checkpoints or immunomodulators, effector cells, and suppressor cells. For each class, a sample-wise Z score from gene expression data was extracted and calculated. The weighted averaged Z score was then calculated by averaging the Z scores within the respective category leading to four values, and the sum of the weighted averaged Z score of the four categories.

### Significantly Mutated Genes

We identified significantly mutated genes (SMG) by using the MutSigCV algorithm (29). MutSigCV measures the significant enrichment of non-silent somatic mutations in a gene by addressing mutational context-specific background mutation rates. Candidate SMGs were required to meet these criteria: statistically significant (*q* < 0.1) and expressed in the human cancer cell lines Encyclopedia (CCLE) (30).

### Deciphering Mutational Signature Operative in the Genome

The R package Maftools proposed by Mayakonda et al. (31) was used to extract mutational signatures from the TCGA genomic data. The ExtractSignatures function based on Bayesian variant non-negative matrix factorization, factorized the mutation portrait matrix into two non-negative matrices “***signatures***” and “***contributions***,” where “***signatures***” represented mutational processes and “***contributions***” represented the corresponding mutational activities. Specifically, the number of columns of matrix “***signatures***” indicated the number of extracted signatures and the rows indicated the 96 mutational contexts (i.e., C > G, C > A, C > T, T > C, T > A, T > G and combined their 5′ and 3′ adjacent bases). The SignatureEnrichment function can automatically determine the optimal number of extracted mutational signatures and assign them to each sample based on the mutational activities. The extracted mutational portrait of CC was compared and annotated by cosine similarity analysis against the Catalogue of Somatic Mutations in Cancer (COSMIC) (32).

### Statistical Analysis

The statistical analysis in this study was generated by R-3.6.1. For quantitative data, statistical significance for comparisons of two groups or more than two groups was estimated by the Wilcoxon rank-sum test or the Kruskal–Wallis H test, respectively. Fisher’s exact test was employed for comparisons of qualitative variables. Logistic regression analysis was used to test the association between TML and risk signature. For the genes with prognostic ability, the Cox proportional hazards model with a Lasso penalty (iteration = 1000) was employed to find the best gene model utilizing the R package “glmnet.” The immune signature was based on the linear combination of the selected mRNA expression level and weighted by their Lasso-Cox regression coefficients. The association between immune signature and prognosis were analyzed by the Cox proportional hazards model and the Kaplan-Meier survival analysis with the R survival package (Survminer 0.4.7). The receiver operating characteristic (ROC) curve was used to assess the prognosis classification performance of the immune risk signature and tumor stage, and the area under the curve (AUC) was compared by DeLong’s test. All comparisons were two-sided with an alpha level of 0.05, and multiple hypothesis testing with the Benjamini-Hochberg method was used to control false discovery rate (FDR) (33).

## Results

### Construction of Immune Risk Signature

**FIGURE 1:**
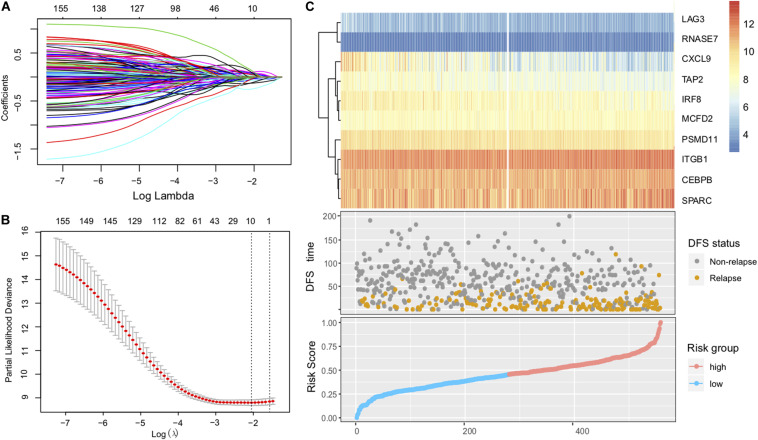
Construction of the immune risk signature model. **(A)** Lasso coefficient profiles of the 161 prognosis-associated immune genes from the discovery (GSE39582 microarray) dataset. **(B)** Partial likelihood deviance of variables revealed by the Lasso regression model. The red dots represented the partial likelihood of deviance values, the gray lines represented the standard error (SE), the two vertical dotted lines on the left and right represented optimal values by minimum criteria and 1-SE criteria, respectively. **(C)** Heatmap of the signature consisting of 10 immune-related genes and the risk score curve based on the Lasso coefficients. Patients were divided into high-risk and low-risk groups and the median risk score was utilized as the cutoff value.

After removing samples without necessary clinicopathological or follow-up data, a total of 1430 CC patients were included for this analysis, including GSE39582 (*N* = 557), GSE17538 (*N* = 200), GSE37892 (*N* = 130), and GSE33113 (*N* = 90), and TCGA (*N* = 453). Considering that the GSE39582 cohort from the Marisa et al. contained the largest sample size (*N* = 557) and included detailed clinical features, we therefore selected it as the discovery dataset to identify the immune risk signature that is associated with CC patients’ prognosis. The univariate analysis was performed in all of the 1508 immune-related genes for the discovery dataset (GSE39582). Through the univariate analysis and log-rank test, a total of 161 genes with prognostic ability were identified (*P* < 0.05). The 161 immune-related genes were then subjected to the Lasso-Cox proportional hazards regression and tenfold cross-validation to generate the best gene model. The Lasso coefficient profile plot was produced against the log(k) sequence, and the minimize k method resulted in 10 optimal coefficients (Figures 1A,B). Finally, a gene model with 10 immune-related genes reached the optimal regression efficiency to speculate the prognostic ability.

The identified immune-related genes included antigen processing and presentation related genes (*LAG3, PSMD11, TAP2*), defense response to infection (*CEBPB, CXCL9, IRF8, RNASE7*), epithelial cell migration (*ITGB1, SPARC*), and *MCFD2*. Furthermore, we constructed an immune risk signature to estimate the risk score of each patient based on the linear combination of the 10 mRNA expression levels weighted by their Lasso-Cox regression coefficients: Immune Risk Score = (0.1979) × *CEBPB* + (−0.2140) × *CXCL9* + (−0.0927) × *IRF8* + (0.5896) × *ITGB1* + (0.2108) × *LAG3* + (0.2489) × *MCFD2* + (−0.2909) × *PSMD11* + (0.5255) × *RNASE7* + (0.0881) × *SPARC* + (−0.1490) × *TAP2* (Figure 1C, Supplementary Table S1). A heatmap of the identified 10-gene expression level and the scatterplot of relapse-free survival (RFS) with corresponding risk score were illustrated in Figure 1C.

### The Prognostic Value of 10-mRNA Immune Signature

**FIGURE 2:**
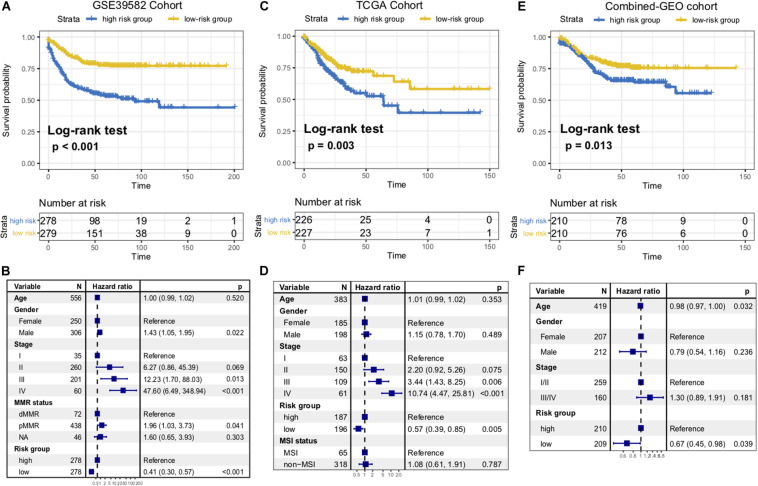
Immune risk signature was associated with CC survival. Kaplan-Meier curves of relapse-free survival according to immune signature groups in the GSE39582 discovery cohort **(A)**, TCGA cohort **(C)**, and another combined-GEO validation cohort **(E)**. Forest plot representation of the multivariate Cox regression model delineated the association between immune risk signature and survival in the three cohorts **(B,D,F)**. Age, gender, clinical stage, or dMMR were taken into account.

To identify the immune signature responsible for CC survival prediction, we divided the discovery cohort samples into a low-risk group (*N* = 279) and a high-risk group (*N* = 278) by using the median risk score as a cutoff point. Patients with low-risk were significantly associated with better RFS compared with those of high-risk (*P* < 0.001, log-rank test; Figure 2A). This association remained markedly significant in the multivariate Cox model after controlling for age, gender, clinical stage, and mismatch repair (MMR) status (HR, 0.41 [95% CI: 0.30–0.57], *P* < 0.001; Figure 2B).

To confirm that the 10-mRNA-based immune signature classifier had similar prognostic value in different populations, we further corroborate this association in the TCGA dataset and another combined-GEO microarray dataset (including GSE17538, GSE33113, and GSE37892; “Materials and Methods” section). Heatmaps of the signature consisting of 10 immune-related genes and the scatterplot of RFS time with corresponding risk score in two external validation cohorts were shown in Supplementary Figure S1. In the TCGA and combined-GEO datasets, we also found that patients with low-risk scores demonstrated a better prognosis than those with high-risk scores (TCGA: *P* = 0.003, Figure 2C; Combined-GEO cohort: *P* = 0.013, Figure 2E; log-rank test). Multivariate Cox proportional hazards regression analysis further revealed that the signature could serve as an independent predictor of patients’ survival outcome after being adjusted for clinicopathologic features in two validation cohorts (TCGA: HR, 0.57 [95%CI, 0.39–0.85], *P* = 0.005, Figure 2D; Combined-GEO cohort: HR, 0.67 [95%CI, 0.45–0.98], *P* = 0.039. Figure 2F). Further analysis confirmed that the higher immune risk signature score was associated with significantly worse tumor staging in CC cohorts (Kruskal–Wallis H test, GSE39582, and TCGA cohorts, both *P* < 0.001, Supplementary Figures S2A,B). Moreover, we found the risk scoring model could improve the accuracy of predictions of survival when combined with the tumor staging system (AUC of GSE39582: Stage vs Risk score + Stage, 67.44 vs 71.56, *P* = 0.002; Risk score vs Risk score + Stage, 67.63 vs 71.56, *P* = 0.071; AUC of TCGA: Stage vs Risk score + Stage, 69.27 vs 72.26, *P* = 0.035; Risk score vs Risk score + Stage, 64.65 vs 72.26, *P* = 0.004; DeLong’s test, Supplementary Figures S2C,D).

### Extracted Immune Risk Signature Associated With Leukocytes Infiltration and Tumor Immunogenicity

**FIGURE 3:**
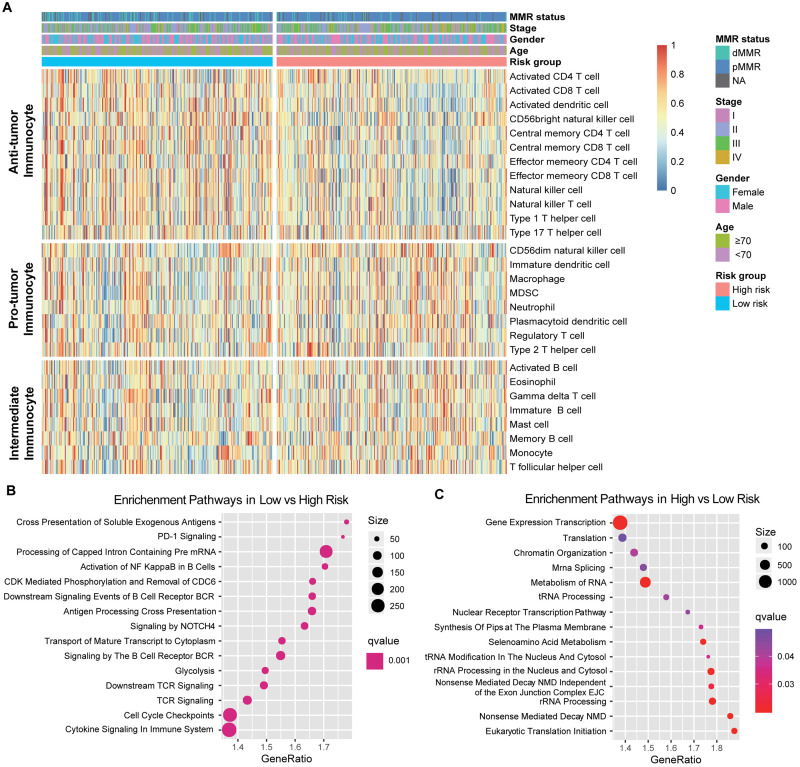
Immune risk signature was associated with the immune infiltration. **(A)** Single-sample gene set enrichment analysis identified the relative infiltration of 28 types of immune cell subpopulations with different risk signature subgroups. The relative infiltration of each cell type was normalized into a Z score. **(B,C)** Top enriched gene pathways in distinct immune risk signature groups (low vs high, left panel; high vs low, right panel) from discovery cohort were assessed by using the GSEA algorithm.

Since the prognosis-related risk signature was extracted from the immune-related genes database, we speculated that its status may regulate the leukocyte infiltration and gene pathways enrichment. Therefore, we composed a heatmap with ssGSEA to visualize the relative abundance of 28 immune infiltrating cell subpopulations from the discovery dataset (Figure 3A). Anti-tumor lymphocyte cell subpopulations, like activated CD4^+^/CD8^+^ T cells, effector memory CD4^+^/CD8^+^ T cells, and natural killer T cells were enriched in the low-risk signature group (*P* < 0.05). Nevertheless, myeloid-derived suppressor cells (MDSC), immature dendritic cells, neutrophils, and regulatory T cells, which belonged to pro-tumor leukocytes, were elevated in the high-risk signature group (*P* < 0.05). We also further characterized the immune infiltration profile in TCGA and the combined-GEO validation cohort, and a similar tendency was observed in these cohorts of such risk signature stratification (Supplementary Figure S3).

Furthermore, GSEA on the CC gene expression profile against REACTOME reference datasets revealed the risk signature related biological signaling pathway. Genes involved in antigen processing cross-presentation, B cell/T cell receptor and immune cytokine signaling pathways were significantly enriched in the low immune risk signature group (Figure 3B, Supplementary Figure S4A). However, chromatin organization and RNA processing and modification were enriched in the high-risk group (Figure 3C, Supplementary Figure S4B).

**FIGURE 4:**
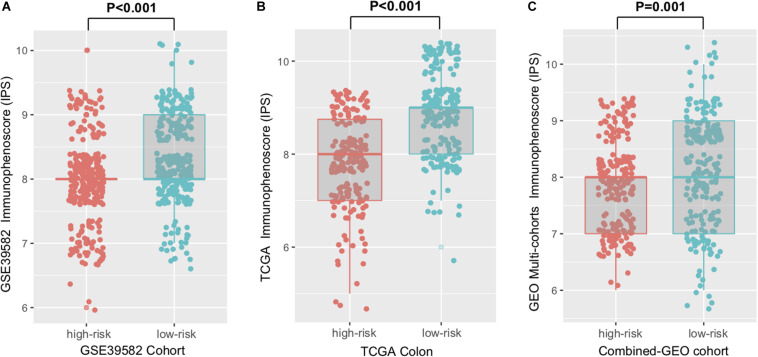
Distribution of immunophenoscore (IPS) in high-risk versus low-risk colon cancer subtypes. Boxplot representation of IPS in the high-risk versus low-risk groups in discovery cohort **(A)**, TCGA cohort **(B)**, and another combined-GEO cohort **(C)**. *P* values as indicated (Wilcoxon rank-sum test).

Immunophenoscore (IPS) was known to determine the tumor immunogenicity and predict response to ICI therapy in multi-types of tumors. Here, we utilized IPS to investigate the relationship between the newly identified signature and immune response. In the discovery cohort, the low-risk signature group had a significantly higher IPS compared with the high-risk group (Wilcoxon rank-sum test, *P* < 0.001, Figure 4A), and this association was also verified in TCGA and the combined-GEO cohort (TCGA, *P* < 0.001, combined-GEO cohort, *P* = 0.001; Figures 4B,C). These findings indicated that CC patients with the immune signature may be more sensitive to ICI treatment.

### Immune Signature Determined the Colon Cancer Genomic Landscape

**FIGURE 5:**
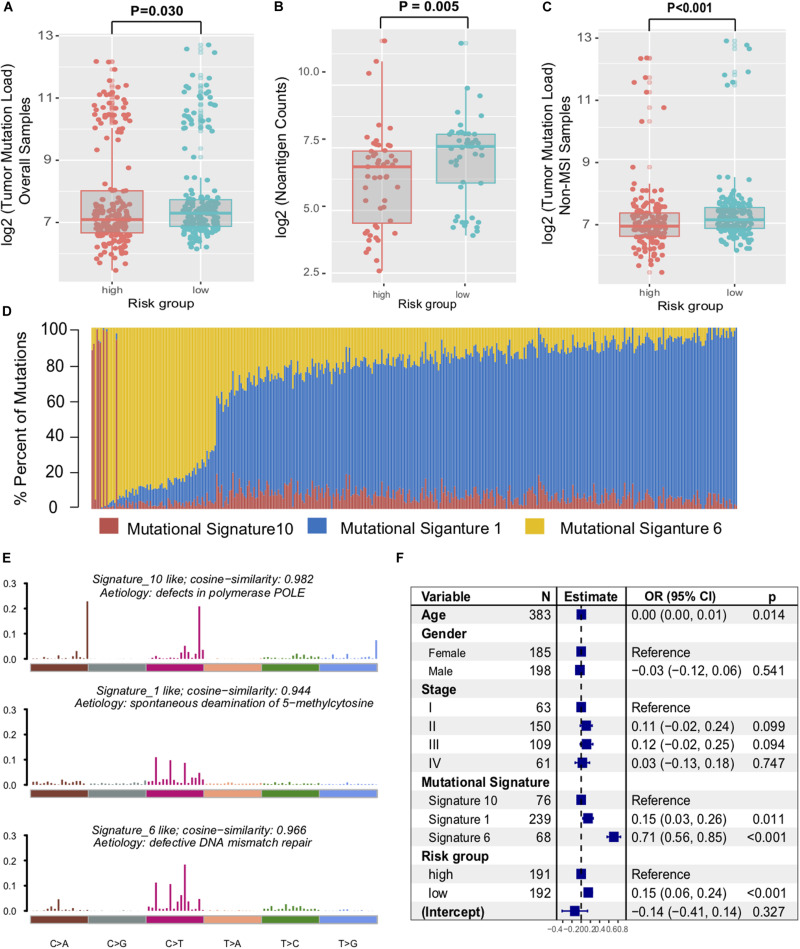
Immune signature was associated with the TML in colon cancer. Tumor mutation load **(A)** and neoantigen counts **(B)** in colon cancer samples were compared with the immune risk signature group. **(C)** Distribution of mutational load in non-MSI samples were also assessed between high-risk and low-risk subtypes. **(D)** Mutational exposures (number of mutations) were attributed to each mutation signature. **(E)** The mutational activities of corresponding extracted mutational signatures (signature 1, 6, and 10, named as COSMIC database). **(F)** Multivariate Logistic regression analysis of TML with respect to immune signature was adjusted by taking into account age, gender, stage, and mutational signatures. *P* values as indicated (Wilcoxon rank-sum test).

Genomic characteristics, such as tumor non-synonymous mutation load (TML) and mutational signatures (e.g., MMR, POLE signature) have shown a strong correlation with clinical response to ICI treatment (34). Therefore, we investigated the association between the immune signature and the genomic mutational landscape. Patients with a low-risk immune signature exhibited a higher mutation load than those with a high-risk signature in the TCGA dataset (*P* = 0.030, Figure 5A). We further compared the tumor neoantigen counts and observed similar results in the group classification (*P* = 0.005, Figure 5B). As high microsatellite instability (MSI-H) tumors accumulated substantial numbers of somatic mutations and significantly affected the TML, we removed the samples with MSI-H and obtained a significantly higher TML in the low-risk signature (*P* < 0.001, Figure 5C).

To gain further insights into the mutational processes operative in CC samples, we extracted the mutational signatures (i.e., signatures 1, 6, 10, Supplementary Figure S5) against the COSMIC database with varying numbers of somatic mutations from the genomic data (Figure 5D). The extracted mutational signatures included defects in DNA proofreading owing to recurrent somatic mutations in POLE (signature 10, 79524 of 264763 [30.0%]), clock-like accumulation of C > T at cytosine-phosphate-guanine dinucleotide (signature 1, 46,106 of 264,763 [17.4%]), and defective MMR (signature 6, 139,133 of 264,763 [52.6%]) (Figure 5E). Hence, mutational counts attributed to signature 6 were significantly higher than other signatures (Kruskal–Wallis H test, *P* = 0.019). To rule out the possibility that associations between immune signature and TML were affected by these confounding factors, we included all mutational signatures and clinical factors in the multivariate logistic regression model. Associations between the immune risk signature and TML remained statistically significant (OR, 0.15 [95% CI, 0.06–0.24], *P* < 0.001, Figure 5F).

**FIGURE 6:**
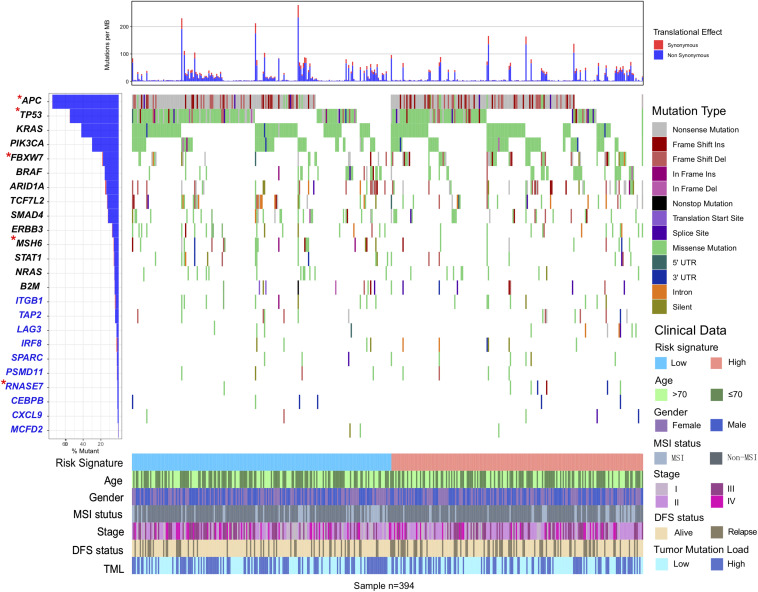
Mutational landscape of SMGs and immune-genes in TCGA COAD cohort stratified by high-risk and low-risk signature groups. The middle panel depicts the mutation relation of SMGs across analyzed cases with mutation types color-coded differently. SMGs with significantly different mutation rates between subgroups were highlighted in upper left asterisk.

We also performed SMG analysis for CC samples in the low-risk versus the high-risk subgroup. The SMG mutational landscapes of these two subgroups (Figure 6) exhibited a distinct mutation ratio in *APC* [138 of 200 (69.0%) vs 154 of 194 (79.4%); *P* = 0.021], *TP53* [111 of 200 (55.5%) vs 83 of 194 (42.8%); *P* = 0.012], *FBXW7* [19 of 200 (9.5%) vs 50 of 194 (25.7%); *P* < 0.001], and *MSH6* [24 of 200 (12.7%) vs 9 of 194 (7%); *P* = 0.010]. The mutation plot of the four SMGs with different immune signature status were shown in Supplementary Figure S6. Besides, we also explored the mutational rate of the aforementioned 10-immune genes, and observed *RNASE7* mutation was enriched in the high-risk subgroup [1 of 200 (0.5%) vs 3 of 194 (3.1%); *P* = 0.014].

### The Immune Risk Signature in the Role of ICI Treatment

**FIGURE 7:**
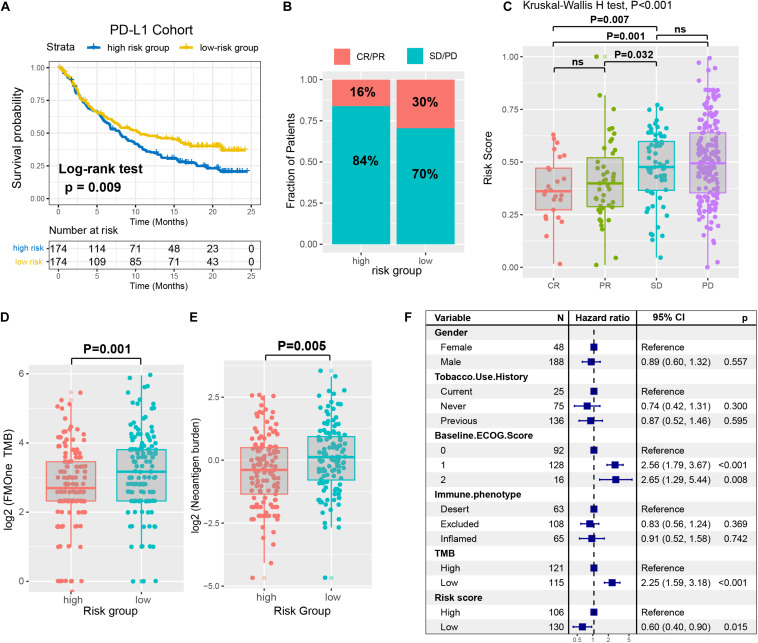
The immune risk signature in the role of ICI treatment. **(A)** Survival analysis of the high versus low immune risk subgroup in the anti-PD-L1 cohort (IMvigor210 cohort) was created using Kaplan-Meier curves. **(B)** The proportion of immune response to anti-PD-L1 treatment in high versus low immune risk score subgroups. CR, complete response; PR, partial response; SD, stable disease; PD, progressive disease. **(C)** Distribution of immune risk score in different immune response statuses. Tumor mutation load **(D)** and neoantigen burden **(E)** in the immunotherapy cohort were compared among distinct immune risk signature subgroups. **(F)** Multivariate Cox regression analysis of immune risk signature with gender, smoking, ECOG score, immunophenotype, and TML status were taken into account. *P* values as indicated (Kruskal–Wallis H test).

Immune checkpoint inhibitors (ICI) therapy represented by anti-PD-1/L1 agents have undoubtedly made a great breakthrough in anti-tumor therapy. Therefore, we curated the gene expression profile and clinical features from an immunotherapy cohort (Imvigor210) of urothelial cancer (UC) treated by anti-PD-L1 agent, so as to investigate the relationship between the constructed risk signature and immune response. In this anti-PD-L1 cohort, patients with a low-risk immune signature score exhibited markedly clinical benefits and a significantly prolonged survival rate (HR, 0.71 [95% CI: 0.55–0.92], *P* = 0.009, Figure 7A). The significant therapeutic advantages and immune response to PD-L1 blockades were observed in samples with a low-risk score compared to those with a high-risk score (Fisher extract test, *P* = 0.008, Figure 7B; Kruskal-Wallis H test, *P* < 0.001, Figure 7C). Further analysis revealed that TML and neoantigen burden were significantly elevated in tumors with low-risk score, which closely linked to immunotherapeutic efficacy (Figures 7D,E). Besides, the association between immune risk score and immunotherapy survival remained statistically significant after taking into account gender, smoking, ECOG score, immunophenotype and, TML status (HR, 0.60 [95%CI, 0.40–0.90], *P* = 0.015; Figure 7F).

## Discussion

Although it has long been recognized that immune contexture plays a vital role in tumor initiation and development (35), these insights have not formed a significant impact on routine clinical application. This highlights the important role of TIM estimation in predicting clinical development and progression of CC patients. In this investigation, we established a reliable prognostic risk signature based on 10 immune-related genes in an independent microarray dataset and proved its efficacy in the TCGA and combined GEO datasets across different platforms. This signature stratified the patients into subgroups with different immune risk, representing distinct tumor immune infiltration level and neoantigen burden. Therefore, the newly identified immune risk signature presumably represented the status of TIM for CC patients and served as a potential biomarker for prognosis estimation and clinical response prediction to immunotherapy.

This study confirmed that the immune risk signature was significantly associated with CC patients’ RFS, and this association remained significant after controlling for clinical-pathological features. More importantly, our signature was based on immune-related genes and revealed a correlation with non-synonymous mutation load and neoantigen counts. Considering the importance of mutational load in predicting the response to anti-PD-1/L1 treatment (12, 36), we speculated that patients with a low-risk immune signature may be more sensitive to ICI therapy. Actually, the IPS and neoantigen load, which determined the tumor immunogenicity and antitumor immune response, also demonstrated a strong connection with this signature. To clarify the effects of this immune signature, we took TML and IPS as the confounding factors into the multivariate Cox regression models, and identified that the immune risk score remained statistically significant in the TCGA cohort (HR, 0.59 [95%CI, 0.39–0.87], *P* = 0.008; Supplementary Figure S7). These findings further indicated its practical implication in precision immunotherapy.

In recent years, numerous studies focused on the immune landscape have brought attention to biological and clinical cancer research. Individual immune cell markers such as CD3^+^ and CD8^+^ T cells have shown prognostic impacts in patients suffering from CC (6). The immune cell subpopulations estimation algorithm (e.g., ssGSEA, CIBERSORT) was frequently utilized to characterize the immune infiltration profiles and analyze the association with clinical therapy (37). Our research also leveraged the aforementioned method and demonstrated enhanced effector T-cells (CD4/CD8^+^ T cell, NK cells), reduced suppressive regulatory T-cells, and MDSC infiltration in low immune risk signature. Meanwhile, signaling pathways involved in the antigen processing and presentation, B cell/T cell receptor and immune cytokine were significantly altered in different risk subgroups, suggested that our signature was a superior prediction determinant of tumor immune infiltration.

Comprehensive knowledge of the mutated driver genes underlying human cancers is a critical foundation for cancer diagnostics, therapeutics, and selection of rational therapies. Here, we used MutSigCV algorithms followed by further filter criteria and identified that SMGs of *APC* and *FBXW7* mutations were enriched in high-risk groups, *TP53* and *MSH6* were enriched in low-risk groups. *APC* was the most common mutational gene in colorectal cancer, and its mutation has indicated a highly significant association with immune resistance (38). *FBXW7* is a critical tumor suppressor of human cancers, missense mutations in this gene show a shorter overall survival rate when compared with wild-type patients in CC (39). *TP53* and *MSH6* mutations may lead to a higher TML owing to the dysregulation of DNA damage repair function (40). Recent research suggested that *TP53* mutations significantly induced the expression of immune checkpoint molecules and activated T-effector and interferon-γ signatures, indicating *TP53* mutation patients would be more sensitive to checkpoint blockade (41).

Nevertheless, there were several limitations in our investigation. The main limitation stemmed from using a public dataset for different cohorts which can be somewhat heterogeneous in data processing and patient population. The risk signature was identified by using retrospective datasets, therefore, the expression profiles of the 10 genes combined with clinical validation in the patients of CC prospective cohort are needed to prove its efficacy. Besides, mutational results derived from the TCGA COAD genomic landscape were not validated in independent datasets owing to the unavailability of mutation data. Finally, due to a lack of CC cohorts being treated by ICIs, we are unable to verify the association between the signature and the immunotherapeutic responsiveness and believe further research is needed.

To summarize, this study identified a new immune risk signature that can not only predict CC patients’ survival outcomes but also represent the immune infiltration status. This signature can be clinically utilized for the improvement of CC patients’ survival, personalize therapy methods based on the risk score, and provide new clues for enrolling CC patients in ICI treatment. However, further randomized control trials are required to validate the significance of the generated signature.

## Data Availability Statement

The datasets generated for this study can be found in the GEO – GSE39582, GSE17538, GSE37892, and GSE33113.

## Author Contributions

HC, WC, and XYL: conception and design. XYL and HC: development of methodology. XYL, DW, XKL, and CY: acquisition of data (provided animals, acquired and managed patients, and provided facilities). XYL, HC, and WC: analysis and interpretation of data (e.g., statistical analysis, biostatistics, and computational analysis). HC and WC: writing, reviewing, and/or revising the manuscript, administrative, technical, or material support (i.e., reporting or organizing data, and constructing databases), and study supervision. All authors contributed to the article and approved the submitted version.

## Conflict of Interest

The authors declare that the research was conducted in the absence of any commercial or financial relationships that could be construed as a potential conflict of interest.

## Footnotes

1 https://www.ncbi.nlm.nih.gov/geo/

2 https://cancergenome.nih.gov/

3 https://immport.niaid.nih.gov

4 https://portal.gdc.cancer.gov/

## Supplementary Material

FIGURE S1 Heatmap of the signature consisting of 10 immune related genes and the risk score curve in the TCGA and combined-GEO colon cancer cohort.

FIGURE S2 Immune risk score associated with tumor stage. Distribution of immune risk score with respect to clinical tumor stage was shown in the GSE39582 **(A)** and TCGA **(B)** cohort. A receiver operating characteristic (ROC) curve was used to assess the prognosis classification performance of the immune risk signature vs tumor stage vs risk signature plus stage in the GSE39582 **(C)** and TCGA **(D)** cohort. The area under the curve (AUC) in different subgroups was calculated by DeLong’s test.

FIGURE S3 Estimation of the relative infiltration of 28 types of immune cell subpopulations with different immune signature groups in the external TCGA and combined-GEO cohort.

FIGURE S4 GSEA enrichment plots show enriched gene sets against REACTOME datasets in low-risk vs high-risk **(A)** and high-risk vs low-risk **(B)**. NES, Normalized Enrichment Score.

FIGURE S5 Mutational signatures extracted from the TCGA COAD genomic dataset. **(A)** The progress of automatically determining the optimal number of mutational signatures (*N* = 3). **(B)** Cosine similarity analysis of extracted mutational signatures against the 30 identified signatures in the Catalog of Somatic Mutations in Cancer (COSMIC, v2) with heatmap illustration.

FIGURE S6 Lollipop plot showing the protein change of four novel SMGs with respect to risk signature in the COAD cohort.

FIGURE S7 Multivariate Cox regression analysis of immune risk signature by taking into account confounding factors, such as age, gender, stage, MSI status, TML, and IPS.

TABLE S1 Lasso Cox regression coefficients of the 10 immune signature genes.

TABLE S2 Clinical characteristics of patients with colon cancer in five datasets.

## References

[1] Siegel RL Miller KD Fedewa SA Ahnen DJ Meester RGS Barzi A Colorectal cancer statistics, 2017. *CA Cancer J Clin.* (2017) 67:177–93. 10.3322/caac.21395 28248415

[2] Schmoll HJ Van Cutsem E Stein A Valentini V Glimelius B Haustermans K ESMO consensus guidelines for management of patients with colon and rectal cancer. A personalized approach to clinical decision making. *Ann Oncol.* (2012) 23:2479–516. 10.1093/annonc/mds236 23012255

[3] Mahajan UM Langhoff E Goni E Costello E Greenhalf W Halloran C Immune cell and stromal signature associated with progression-free survival of patients with resected pancreatic ductal adenocarcinoma. *Gastroenterology.* (2018) 155:1625–39.e2. 10.1053/j.gastro.2018.08.009 30092175

[4] Jia Q Wu W Wang Y Alexander PB Sun C Gong Z Local mutational diversity drives intratumoral immune heterogeneity in non-small cell lung cancer. *Nat Commun.* (2018) 9:5361. 10.1038/s41467-018-07767-w 30560866 PMC6299138

[5] Souza P Rizzardi F Noleto G Atanazio M Bianchi O Parra ER Refractory remodeling of the microenvironment by abnormal type V collagen, apoptosis, and immune response in non-small cell lung cancer. *Hum Pathol.* (2010) 41:239–48. 10.1016/j.humpath.2009.07.018 19828174

[6] Galon J Costes A Sanchez-Cabo F Kirilovsky A Mlecnik B Lagorce-Pages C Type, density, and location of immune cells within human colorectal tumors predict clinical outcome. *Science.* (2006) 313:1960–4. 10.1126/science.1129139 17008531

[7] Mlecnik B Tosolini M Kirilovsky A Berger A Bindea G Meatchi T Histopathologic-based prognostic factors of colorectal cancers are associated with the state of the local immune reaction. *J Clin Oncol.* (2011) 29:610–8. 10.1200/JCO.2010.30.5425 21245428

[8] Pages F Mlecnik B Marliot F Bindea G Ou FS Bifulco C International validation of the consensus immunoscore for the classification of colon cancer: a prognostic and accuracy study. *Lancet.* (2018) 391:2128–39. 10.1016/S0140-6736(18)30789-X 29754777

[9] Le DT Durham JN Smith KN Wang H Bartlett BR Aulakh LK Mismatch repair deficiency predicts response of solid tumors to PD-1 blockade. *Science.* (2017) 357:409–13. 10.1126/science.aan6733 28596308 PMC5576142

[10] Xiao Y Freeman GJ. The microsatellite instable subset of colorectal cancer is a particularly good candidate for checkpoint blockade immunotherapy. *Cancer Discov.* (2015) 5:16–8. 10.1158/2159-8290.CD-14-1397 25583798 PMC4295637

[11] Ruiz-Banobre J Goel A. DNA mismatch repair deficiency and immune checkpoint inhibitors in gastrointestinal cancers. *Gastroenterology.* (2019) 156:890–903. 10.1053/j.gastro.2018.11.071 30578781 PMC6409193

[12] Samstein RM Lee CH Shoushtari AN Hellmann MD Shen R Janjigian YY Tumor mutational load predicts survival after immunotherapy across multiple cancer types. *Nat Genet.* (2019) 51:202–6. 10.1038/s41588-018-0312-8 30643254 PMC6365097

[13] Llosa NJ Cruise M Tam A Wicks EC Hechenbleikner EM Taube JM The vigorous immune microenvironment of microsatellite instable colon cancer is balanced by multiple counter-inhibitory checkpoints. *Cancer Discov.* (2015) 5:43–51. 10.1158/2159-8290.CD-14-0863 25358689 PMC4293246

[14] Finotello F Trajanoski Z. Quantifying tumor-infiltrating immune cells from transcriptomics data. *Cancer Immunol Immunother.* (2018) 67:1031–40. 10.1007/s00262-018-2150-z 29541787 PMC6006237

[15] Bhattacharya S Andorf S Gomes L Dunn P Schaefer H Pontius J ImmPort: disseminating data to the public for the future of immunology. *Immunol Res.* (2014) 58:234–9. 10.1007/s12026-014-8516-1 24791905

[16] Dai W Li Y Mo S Feng Y Zhang L Xu Y A robust gene signature for the prediction of early relapse in stage I-III colon cancer. *Mol Oncol.* (2018) 12:463–75. 10.1002/1878-0261.12175 29377588 PMC5891048

[17] Marisa L de Reynies A Duval A Selves J Gaub MP Vescovo L Gene expression classification of colon cancer into molecular subtypes: characterization, validation, and prognostic value. *PLoS Med.* (2013) 10:e1001453. 10.1371/journal.pmed.1001453 23700391 PMC3660251

[18] Smith JJ Deane NG Wu F Merchant NB Zhang B Jiang A Experimentally derived metastasis gene expression profile predicts recurrence and death in patients with colon cancer. *Gastroenterology.* (2010) 138:958–68. 10.1053/j.gastro.2009.11.005 19914252 PMC3388775

[19] Laibe S Lagarde A Ferrari A Monges G Birnbaum D Olschwang S A seven-gene signature aggregates a subgroup of stage II colon cancers with stage III. *OMICS.* (2012) 16:560–5. 10.1089/omi.2012.0039 22917480

[20] de Sousa EMF Colak S Buikhuisen J Koster J Cameron K de Jong JH Methylation of cancer-stem-cell-associated Wnt target genes predicts poor prognosis in colorectal cancer patients. *Cell Stem Cell.* (2011) 9:476–85. 10.1016/j.stem.2011.10.008 22056143

[21] Jorissen RN Gibbs P Christie M Prakash S Lipton L Desai J Metastasis-associated gene expression changes predict poor outcomes in patients with dukes stage B and C colorectal cancer. *Clin Cancer Res.* (2009) 15:7642–51. 10.1158/1078-0432.CCR-09-1431 19996206 PMC2920750

[22] Mariathasan S Turley SJ Nickles D Castiglioni A Yuen K Wang Y TGFbeta attenuates tumour response to PD-L1 blockade by contributing to exclusion of T cells. *Nature.* (2018) 554:544–8. 10.1038/nature25501 29443960 PMC6028240

[23] Rooney MS Shukla SA Wu CJ Getz G Hacohen N. Molecular and genetic properties of tumors associated with local immune cytolytic activity. *Cell.* (2015) 160:48–61. 10.1016/j.cell.2014.12.033 25594174 PMC4856474

[24] Chen H Yang M Wang Q Song F Li X Chen K. The new identified biomarkers determine sensitivity to immune check-point blockade therapies in melanoma. *Oncoimmunology.* (2019) 8:1608132. 10.1080/2162402X.2019.1608132 31413919 PMC6682357

[25] Ritchie ME Phipson B Wu D Hu Y Law CW Shi W limma powers differential expression analyses for RNA-sequencing and microarray studies. *Nucleic Acids Res.* (2015) 43:e47. 10.1093/nar/gkv007 25605792 PMC4402510

[26] Subramanian A Tamayo P Mootha VK Mukherjee S Ebert BL Gillette MA Gene set enrichment analysis: a knowledge-based approach for interpreting genome-wide expression profiles. *Proc Natl Acad Sci USA.* (2005) 102:15545–50. 10.1073/pnas.0506580102 16199517 PMC1239896

[27] Barbie DA Tamayo P Boehm JS Kim SY Moody SE Dunn IF Systematic RNA interference reveals that oncogenic KRAS-driven cancers require TBK1. *Nature.* (2009) 462:108–12. 10.1038/nature08460 19847166 PMC2783335

[28] Charoentong P Finotello F Angelova M Mayer C Efremova M Rieder D Pan-cancer immunogenomic analyses reveal genotype-immunophenotype relationships and predictors of response to checkpoint blockade. *Cell Rep.* (2017) 18:248–62. 10.1016/j.celrep.2016.12.019 28052254

[29] Lawrence MS Stojanov P Polak P Kryukov GV Cibulskis K Sivachenko A Mutational heterogeneity in cancer and the search for new cancer-associated genes. *Nature.* (2013) 499:214–8. 10.1038/nature12213 23770567 PMC3919509

[30] Klijn C Durinck S Stawiski EW Haverty PM Jiang Z Liu H A comprehensive transcriptional portrait of human cancer cell lines. *Nat Biotechnol.* (2015) 33:306–12. 10.1038/nbt.3080 25485619

[31] Mayakonda A Lin DC Assenov Y Plass C Koeffler HP. Maftools: efficient and comprehensive analysis of somatic variants in cancer. *Genome Res.* (2018) 28:1747–56. 10.1101/gr.239244.118 30341162 PMC6211645

[32] Kandoth C McLellan MD Vandin F Ye K Niu B Lu C Mutational landscape and significance across 12 major cancer types. *Nature.* (2013) 502:333–9. 10.1038/nature12634 24132290 PMC3927368

[33] Love MI Huber W Anders S. Moderated estimation of fold change and dispersion for RNA-seq data with DESeq2. *Genome Biol.* (2014) 15:550. 10.1186/s13059-014-0550-8 25516281 PMC4302049

[34] Mandal R Samstein RM Lee KW Havel JJ Wang H Krishna C Genetic diversity of tumors with mismatch repair deficiency influences anti-PD-1 immunotherapy response. *Science.* (2019) 364:485–91. 10.1126/science.aau0447 31048490 PMC6685207

[35] Fridman WH Pages F Sautes-Fridman C Galon J. The immune contexture in human tumours: impact on clinical outcome. *Nat Rev Cancer.* (2012) 12:298–306. 10.1038/nrc3245 22419253

[36] Chen H Chong W Wu Q Yao Y Mao M Wang X. Association of LRP1B mutation with tumor mutation burden and outcomes in melanoma and non-small cell lung cancer patients treated with immune check-point blockades. *Front Immunol.* (2019) 10:1113. 10.3389/fimmu.2019.01113 31164891 PMC6536574

[37] Zhou R Zhang J Zeng D Sun H Rong X Shi M Immune cell infiltration as a biomarker for the diagnosis and prognosis of stage I-III colon cancer. *Cancer Immunol Immunother.* (2019) 68:433–42. 10.1007/s00262-018-2289-7 30564892 PMC6426802

[38] Cristescu R Mogg R Ayers M Albright A Murphy E Yearley J Pan-tumor genomic biomarkers for PD-1 checkpoint blockade-based immunotherapy. *Science.* (2018) 362:eaar3593. 10.1126/science.aar3593 30309915 PMC6718162

[39] Yeh CH Bellon M Nicot C. FBXW7: a critical tumor suppressor of human cancers. *Mol Cancer.* (2018) 17:115. 10.1186/s12943-018-0857-2 30086763 PMC6081812

[40] Gelsomino F Barbolini M Spallanzani A Pugliese G Cascinu S. The evolving role of microsatellite instability in colorectal cancer: a review. *Cancer Treat Rev.* (2016) 51:19–26. 10.1016/j.ctrv.2016.10.005 27838401

[41] Dong ZY Zhong WZ Zhang XC Su J Xie Z Liu SY Potential predictive value of TP53 and KRAS mutation status for response to PD-1 blockade immunotherapy in lung adenocarcinoma. *Clin Cancer Res.* (2017) 23:3012–24. 10.1158/1078-0432.CCR-16-2554 28039262

