# Supplementary material for: Identification of an Immune Signature Predicting Prognosis Risk and Lymphocyte Infiltration in Colon Cancer
Source: Front Immunol. 2020 Sep 3;11:1678. doi: 10.3389/fimmu.2020.01678 (PMC7497441; doi:10.3389/fimmu.2020.01678)
Supplement: FIGURE S1 — Heatmap of the signature consisting of 10 immune related genes and the risk score curve in the TCGA and combined-GEO colon cancer cohort. [file Data_Sheet_1.docx]

| **Supplemental Table S1. Clinical characteristics of patients with colon cancer in five datasets** | | | | | |
| --- | --- | --- | --- | --- | --- |
|  |  |  |  |  |  |
| **Characteristics** | **GSE39582 dataset** | **TCGA dataset** | **GSE17538 dataset** | **GSE33113 dataset** | **GSE37892 dataset** |
| No. of patients | 557 | 453 | 200 | 90 | 130 |
| **Age (years)** |  |  |  |  |  |
| Range | 22-97 | 31-90 | 23-94 | 34-95 | 22-97 |
| Median | 68.1 | 67 | 66 | 73 | 68 |
| **Gender** |  |  |  |  |  |
| Female | 250 | 214 | 98 | 48 | 61 |
| Male | 307 | 239 | 102 | 42 | 69 |
| **Stage** |  |  |  |  |  |
| I/II | 296 | 250 | 98 | 90 | 73 |
| III/IV | 261 | 192 | 102 | 0 | 57 |
| Unknown | 0 | 11 | 0 | 0 | 0 |
| **MSI/MMR status** |  |  |  |  |  |
| Yes | 72 | 66 |  |  |  |
| No | 439 | 328 |  |  |  |
| Unknown | 46 | 59 | 200 | 90 | 130 |
| **Recurrence** |  |  |  |  |  |
| Yes | 177 | 122 | 55 | 19 | 37 |
| No | 380 | 331 | 145 | 71 | 93 |
| **Recurrence-free survival (months)** |  |  |  |  |  |
| Range | 0-201 | 0-150 | 0-142.6 | 1.7-120.0 | 0.6-104.6 |
| Median | 43 | 19.3 | 39 | 39.3 | 43.5 |

**Supplemental Table S2. Lasso Cox regression coefficients of the 10 immune signature genes.**

| **Gene** | **coefficients** |
| --- | --- |
| CEBPB | 0.19785238 |
| CXCL9 | -0.214016776 |
| IRF8 | -0.092682252 |
| ITGB1 | 0.589595739 |
| LAG3 | 0.210754425 |
| MCFD2 | 0.248944777 |
| PSMD11 | -0.290928549 |
| RNASE7 | 0.525490449 |
| SPARC | 0.088119478 |
| TAP2 | -0.149012449 |

**
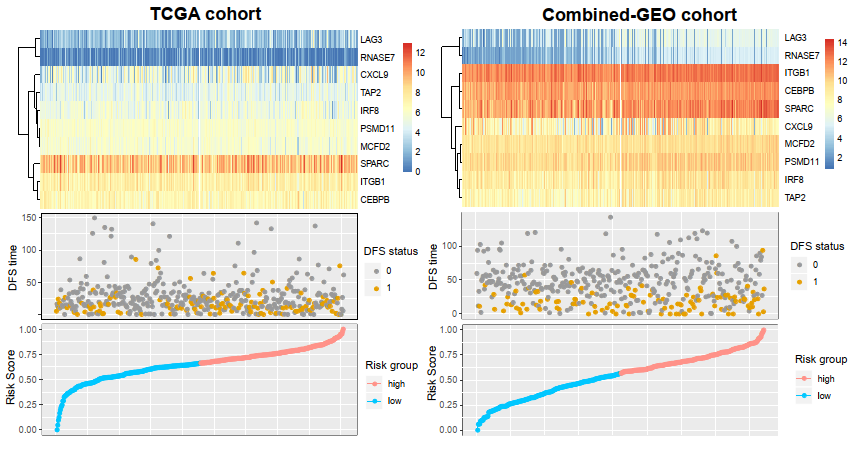
**

**Supplemental Figure S1. Heatmap of the signature consisting of 10 immune related genes and the risk score curve in TCGA and combined-GEO colon cancer cohort.**

**
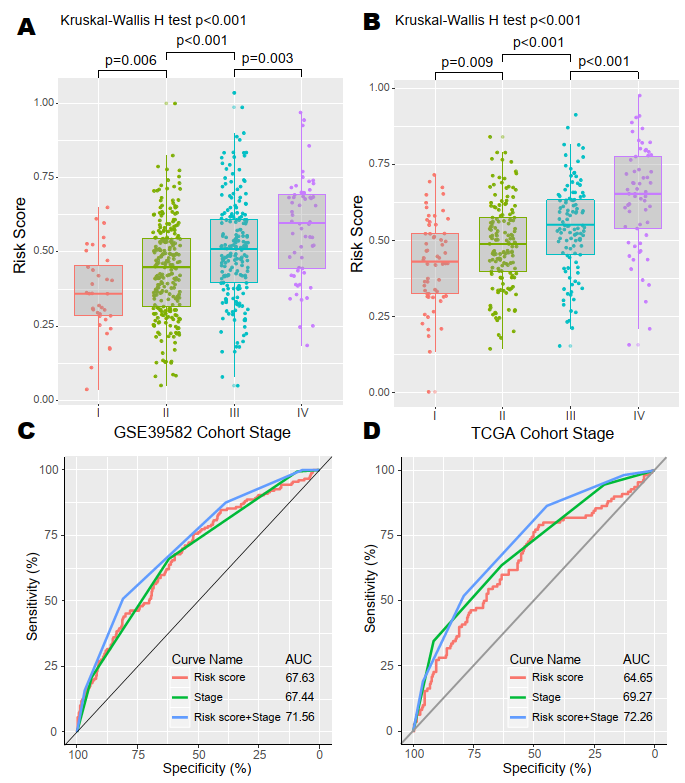
**

**Supplemental Figure S2. Immune risk score associated with tumor stage**. Distribution of immune risk score with respect to clincal tumor stage was shown in GSE39582 (A) and TCGA (B) cohort. Receiver operating characteristic (ROC) curve was used to assess the prognosis classification performance of the immune risk signature vs tumor stage vs risk signature plus stage in GSE39582 (C) and TCGA (D) cohort . Area under of curve(AUC) in different subgroup was calculated by DeLong's test.

**
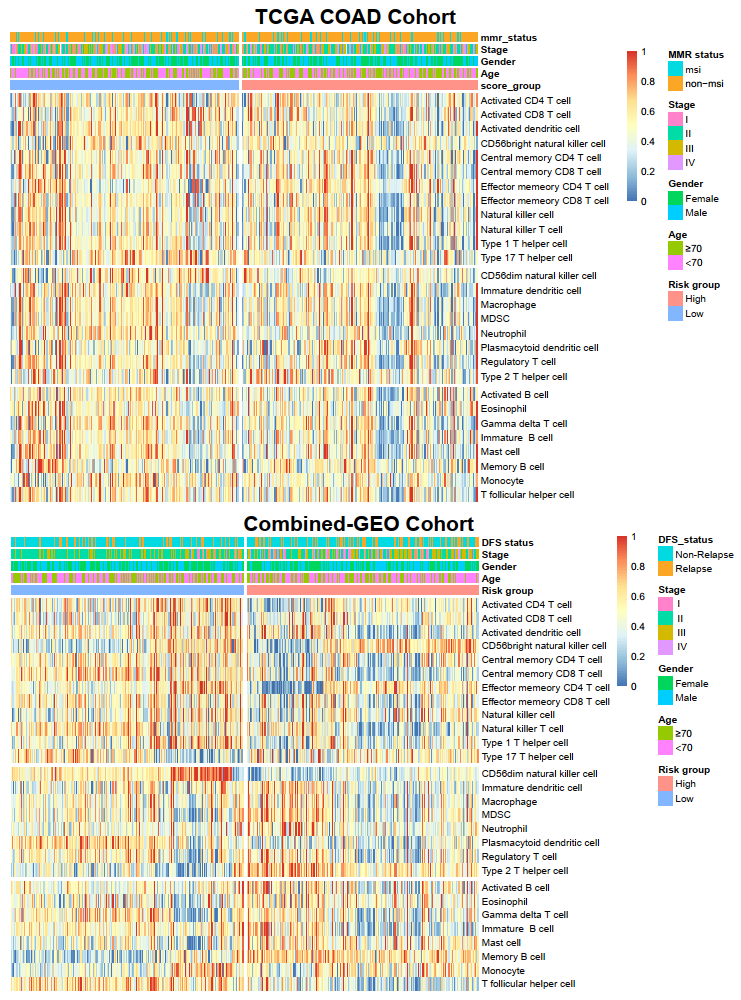
**

**Supplemental Figure S3. Estimation of the relative infiltration of 28 types immune cell subpopulations with different immune signature groups in external TCGA and combined-GEO cohort.**

**
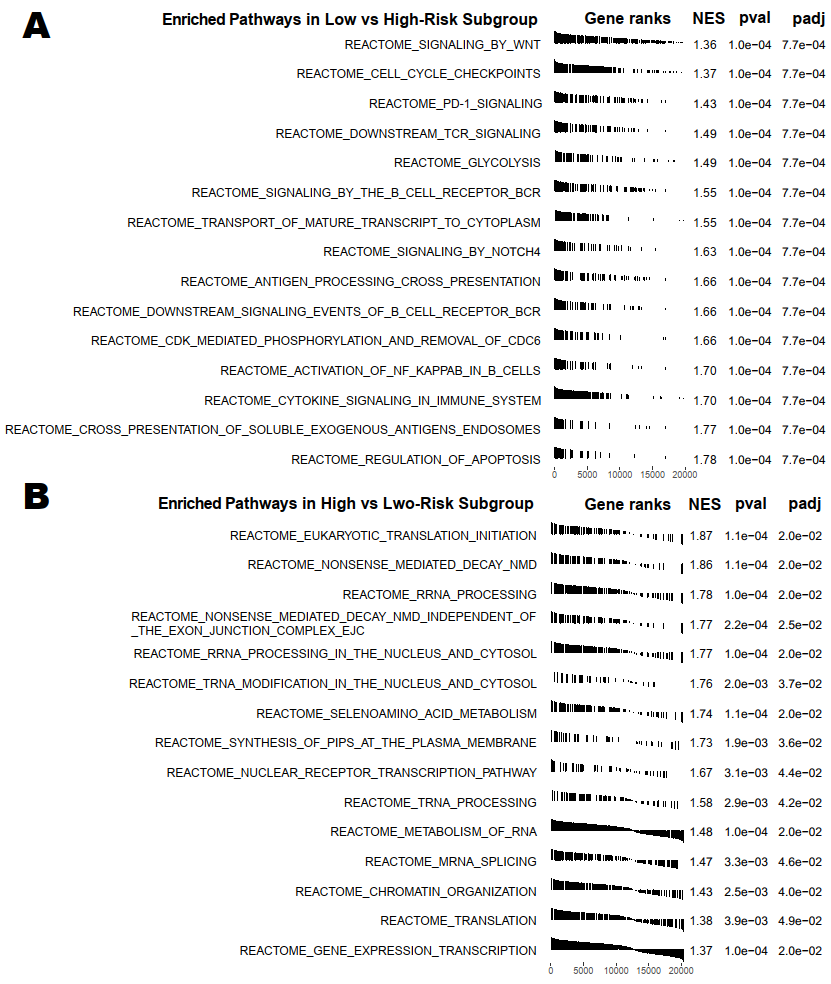
**

**Supplemental Figure S4. GSEA enrichment plots shown enriched gene sets against to REACTOME datasets in** **low-risk vs high-risk (A) and high-risk vs low-risk (B).** NES, Normalized Enrichment Score.

**
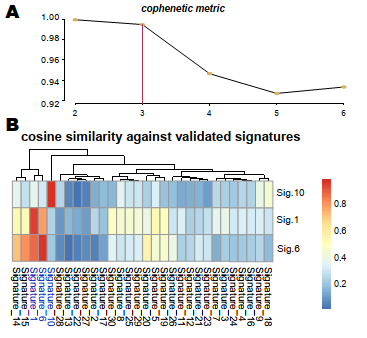
**

**Supplemental Figure S5.** **Mutational signatures extracted from the TCGA COAD genomic dataset.** (A) The progress of automatically determine the optimal number of mutational signatures (N=3). (B) Cosine similarity analysis of extracted mutational signatures against the 30 identified signatures in Catalogue of Somatic Mutations in Cancer (COSMIC, v2) with heatmap illustration.


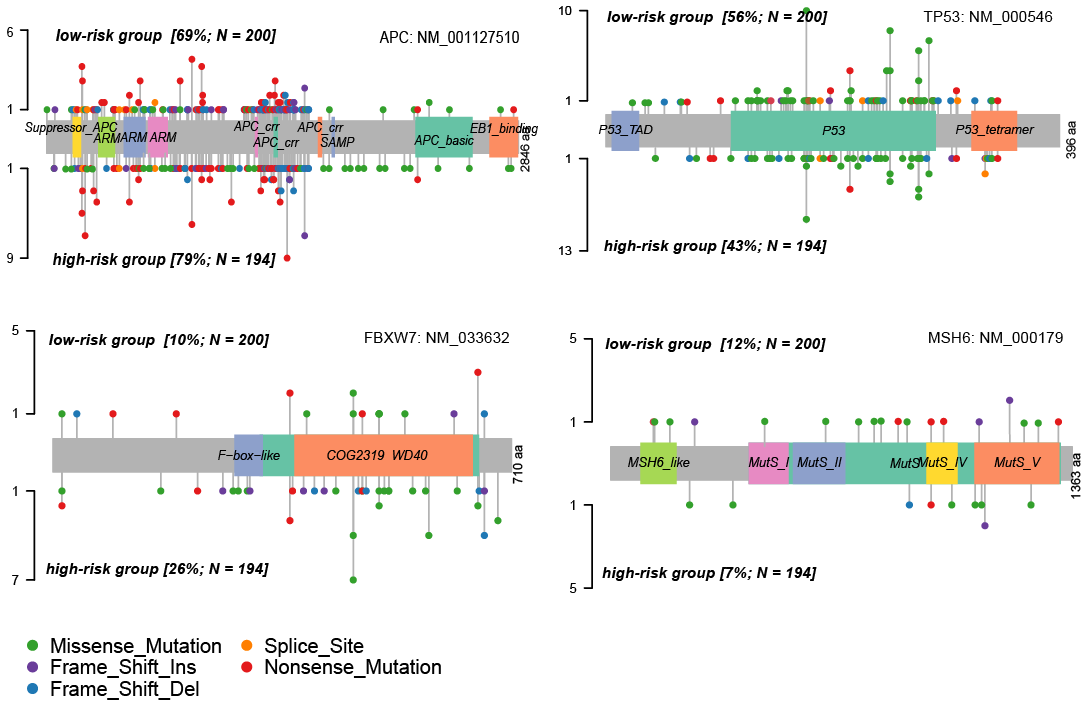


**Supplemental Figure S6. Lollipop plot showed the protein change of four novel SMGs with respect to risk signature in COAD cohort.**


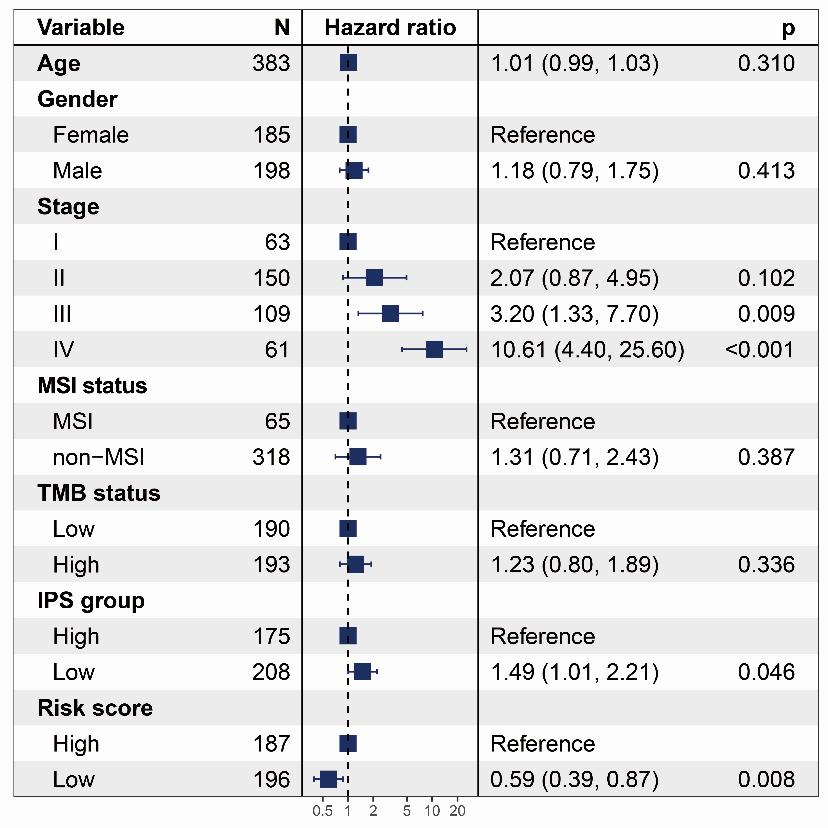


**Supplemental Figure S7. Multivariate Cox regression analysis of immune risk signature by taking into account confounding factors, such as age, gender, stage, MSI status, TML and IPS.**
